# E-selectin gene polymorphisms are associated with essential hypertension: a case-control pilot study in a Chinese population

**DOI:** 10.1186/1471-2350-11-127

**Published:** 2010-08-27

**Authors:** Zuoguang Wang, Ya Liu, Jieling Liu, Kuo Liu, Yuqin Lou, Jie Wen, Qiuli Niu, Shaojun Wen, Zhaosu Wu

**Affiliations:** 1 Department of Hypertension, Beijing Anzhen Hospital, Attached to Capital University of Medical Sciences, Beijing Institute of Heart, Lung, Blood Vessel Diseases, Beijing, 100029, China; 2 Department of Cardiovascular Epidemiology, Beijing Anzhen Hospital, Attached to Capital University of Medical Sciences, Beijing Institute of Heart, Lung, Blood Vessel Diseases, Beijing, 100029, China

## Abstract

**Background:**

Genetic variation is thought to contribute to the etiology of hypertension, and E-selectin is a candidate essential hypertension-associated gene. This study thus sought to investigate possible genetic associations between the T1880C, C602A and T1559C polymorphisms of E-selectin and essential hypertension.

**Methods:**

Hypertensive patients (n = 490) and healthy normotensive subjects (n = 495) were screened for the genotypes T1880C, C602A and T1559C using real-time quantitative polymerase chain reaction after DNA extraction to identify representative variations in the E-selectin gene. The associations between genotypes and alleles of the three mutations and essential hypertension were then analyzed using a case-control study.

**Results:**

Hypertensive patients and normotensive subjects were significantly different with respect to the genotypes CC, CA and AA (P = 0.005) and the C-allele frequency of C602A (P = 0.001). A comparison of dominant versus recessive models also revealed significant differences between the two groups (P = 0.004 and P = 0.02). When subgrouped by gender, these indexes differed significantly between normotensive and essential hypertensive males, but not in females. The additive model of the T1559C genotype did not differ between essential hypertensive and normotensive groups overall (P = 0.39), but it was different between hypertensive and normotensive males (P = 0.046) and females (P = 0.045). The CC + TC versus TT frequency of T1559C was also different in the recessive model of male hypertensive and normotensive groups (P = 0.02). Further analysis showed that C602A and T1559C were significantly associated with hypertension (C602A: OR = 7.58, 95%CI = 1.53-11.97, P < 0.01; and T1559C: OR = 6.77, 95%CI = 1.07-1.83, P < 0.05). The frequency of the C-C-C haplotype was significantly higher in hypertensive patients than in control individuals as well as in hypertensive and normotensive males (P = 0.008 and 0.01). The frequency of the C-A-T haplotype was higher only in male hypertensives and normotensives (P = 0.015). Furthermore, there was a significant interaction between E-selectin and gender (P = 0.02 for C602A and 0.04 for T1559C).

**Conclusion:**

C602A and T1559C may be independent risk factors for essential hypertension in the Chinese population, whereas T1880C is not.

## Background

E-selectin (SELE, CD62E, ELAM-1), an 11-kD cell surface glycoprotein, is an adhesion molecule of the selectin family that mediates the interaction of circulating leukocytes with the vascular endothelium in various physiological and pathological settings [1]. Its corresponding gene *SELE *is located on human chromosome 1. Unique among other selectin family molecules, E-selectin typically is not detected in inactivated endothelial cells, but is rapidly synthesized in response to certain cytokines and other pro-inflammatory stimuli, making it a marker of the "activated" endothelial phenotype [2]. Leukocyte-endothelial interactions contribute to a variety of vascular disease processes, such as acute and chronic inflammation and atherosclerosis. Several soluble factors (e.g., cytokines, chemokines, and growth factors), as well as cell surface adhesion molecules, which are expressed by both endothelial cells and leukocytes, interact in a complex fashion to efficiently mediate leukocyte recruitment [3,4].

Several recent findings regarding the genetic background of atherosclerosis patients have indicated that DNA polymorphisms in genes that encode adhesion molecules may be associated with a high risk of severe atherosclerosis [5,6]. Moreover, recent studies in a German population showed possible associations between the S128R and L554F variations of the E-selectin gene and severe atherosclerosis, hypertension and cerebrovascular diseases [7,8].

Because E-selectin is related to inflammation and atherosclerosis, and essential hypertension is characterized by chronic inflammation and atherosclerosis, we attempted to explore the association between E-selectin single nucleotide polymorphisms (SNPs) and hypertension. Three candidate SNPs of E-selectin involved in the pathogenesis of inflammation and atherosclerosis--T1880C, C602A, T1559C--were selected a priori on the basis of a genetic analysis, a literature review [9,10], predictive analyses with an emphasis on functionally important variants, and expert opinion. These variants may provide clues to the pathogenesis of essential hypertension.

## Methods

### Study population

All normotensive participants and hypertensive patients were screened at the physical examination center and hypertension clinic at Anzhen Hospital. A total of 495 healthy, normotensive subjects (NT group) and 490 hypertensive patients (EH group) were screened. Blood pressure (BP) was accurately measured three times on different days with a mercury sphygmomanometer by experienced internists in their office. Measurements were taken after the patients had been seated on a chair with their feet on the floor and their arms supported at heart level for 10 min. The definition of normotension (SBP < 130 mmHg and DBP < 85 mmHg) and hypertension (140 ≤ SBP ≤ 179 mmHg, or 90 ≤ DBP ≤ 109 mmHg) were based on the BP classification of the seventh report of the Joint National Committee on Prevention, Detection, Evaluation, and Treatment of High Blood Pressure (JNC-VII). All hypertensive patients were diagnosed as having essential hypertension and had never been treated with any antihypertensive drugs.

No hypertensive patients were suffering from any known diseases, including secondary hypertension, diabetic disease or kidney diseases, that might affect BP. BP was measured according to JNC-VII standards. This study complied with the Declaration of Helsinki. All participants signed a consent form, and the study was approved by the Hospital Ethics Committee [11].

### DNA preparation

With the subject in the sitting position, a 5-mL peripheral venous blood sample was drawn into an EDTA-Na anti-coagulated vacutainer tube with minimal use of a tourniquet. DNA was extracted using the PURGENE kit from Gentra Systems (Minneapolis, MN, USA) and stored at -20°C in aliquots until required.

### Detection of genotype

The C_11975323_20 (T1880C) SNP genotyping kit was obtained from Applied Biosystems. The kit contained two flanking primers as well as C- and T-specific probes labeled with VIC and FAM fluorescent dyes, respectively. Genotyping reactions contained 1× TaqMan^® ^PCR Master Mix, No AmpErase^® ^UNG, and approximately 5 ng of genomic DNA in a final volume of 5 μL. Amplifications were performed using a 7300 Real-Time PCR System and the following thermocycling conditions: initial denaturation and activation at 95°C for 10 minutes, followed by 40 cycles of 95°C for 15 seconds and 60°C for 1 minute. The genotypes C602A and T1559C were detected using the SNP kits C_11975332_10 and C_8919523_1 (Applied Biosystems) following the instructions supplied by the manufacturer.

### Statistics

Values were expressed as means ± SD. The frequency distribution of haplotypes and the association of genotypes with hypertension were analyzed using a chi-squared test. Comparisons of some indexes between the EH and NT groups, including SBP, DBP and BMI, were made using Students' t-test. To test for an association between each SNP and hypertension, we computed the overall genotypic test of association and the three genetic models (dominant, additive, and recessive). A multinomial logistic regression was used to study the effect of E-selectin T1880C, C602A and T1559C variants on hypertension status to allow incorporation of other variables into the model. All tests of association were adjusted for age and gender. Analyses of case-control-biased haplotype, linkage disequilibrium, and Hardy-Weinberg equilibrium were performed using SNPAlyze, version 7.0 Pro (Dynacom Co., Ltd, Mobara, Japan). All analyses were performed using SPSS v.11.5 (SPSS Inc., Chicago, USA) statistical analysis software. A two-tailed P-value < 0.05 was considered to be statistically significant [12].

## Results

Genomic locations and related mapping data (Table 1) were obtained from the National Center for Biotechnology Information (NCBI). The basic clinical characteristics of NT and EH groups are listed in Table 2. Age and sex were matched in the essential hypertensive (EH) and normotensive (NT) groups; body mass index (BMI), total cholesterol (TC), low-density lipoprotein cholesterol (LDL-C), systolic blood pressure (SBP) and diastolic blood pressure (DBP) were not, but these differences did not affect the results of analysis. The expected and observed genotypic frequencies of each SNP were in good agreement with the predicted Hardy-Weinberg equilibrium values (data not shown).

**Table 1:** Location of SNPs analyzed in the present case-control study

dbSNP rs#cluster ID	Contig position	Region	Function	dbSNP allele	Protein residue
T1880C/rs5355	20186225	Exon_13	missense	T/C	Phe/Leu
C602A/rs5361	20191415	Exon_4	missense	C/A	Tyr/His
T1559C/rs5368	20187301	Exon_9	missense	T/C	Arg/Ser

**Table 2:** Clinical characteristics of normotensive and essential hypertensive participants

Index	Total			Male			Female		
			
	NT(n = 495)	EH(n = 490)	P	NT(n = 293)	EH(n = 316)	P	NT(n = 202)	EH(n = 174)	P
**Age**	51.50 ± 8.92	53.75 ± 7.96	0.85	54.72 ± 5.32	52.1 ± 7.64	0.76	51.50 ± 7.91	52.69 ± 6.88	0.91
**BMI (kg/m**^**2**^**)**	22.12 ± 3.12	26.41 ± 3.36	<0.05*	22.1 ± 2.6	24.4 ± 2. 2	0.58	23.9 ± 2.7	27.63 ± 3.05	0.03*
**SBP (mmHg)**	114.38 ± 11.89	140.29 ± 17.68	<0.01**	106.6 ± 23. 5	142. 4 ± 20. 7	<0.01**	104. 5 ± 24. 1	145.63 ± 19.42	<0.01**
**DBP (mmHg)**	75.28 ± 9.10	90.44 ± 12.44	<0.01**	80.6 ± 3.7	90.1 ± 10.4	0.01*	72.5 ± 9.6	94.87 ± 11.47	<0.01**
**TG(mmol/L)**	1.91 ± 0.50	2.03 ± 0.63	0.63	1.07 ± 0. 62	1. 12 ± 0.59	0.72	1.10 ± 0.65	1.32 ± 0.55	0.59
**TC(mmol/L)**	4.88 ± 1.01	5.38 ± 1.73	<0.05*	4.51 ± 1.22	4.59 ± 1.21	0.93	4.64 ± 1.17	5.36 ± 1.08	<0.05*
**HDL-C(mmol/L)**	1.62 ± 0.50	1.71 ± 0.61	0.67	0.96 ± 0.73	1.01 ± 0. 32	0.71	1.79 ± 0.31	1.63 ± 0.42	0.64
**LDL-C(mmol/L)**	1.91 ± 1.79	2.90 ± 1.63	<0.01**	2.08 ± 1.46	2.40 ± 1.48	0.05	2.27 ± 1.41	3.18 ± 1.25	<0.01**
**Glu(mmol/L)**	4.61 ± 1.45	4.98 ± 1.48	0.78	4.30 ± 1.52	4.40 ± 1.41	0.91	4.60 ± 1.39	4.67 ± 1.74	0.95
**BUN(mmol/L)**	5.03 ± 1.17	5.89 ± 1.39	0.55	5.08 ± 1.11	6.12 ± 1. 09	0.52	5.02 ± 1.04	5.34 ± 1.22	0.68
**Cr(μmol/L)**	89.25 ± 15.66	92.05 ± 14.22	0.83	86.23 ± 11.98	90.83 ± 12.12	0.73	87.87 ± 15.57	93.45 ± 12.78	0.54
**UA(μmol/L)**	86.72 ± 22.46	92.34 ± 17.01	0.69	85.60 ± 20.99	91.48 ± 19.25	0.60	85.79 ± 19.42	90.16 ± 18.77	0.61

All the data were presented as mean ± SD. EH, essential hypertensive patients; NT, normotensive subjects; BMI, body mass index; SBP, systolic blood pressure; DBP, diastolic blood pressure; TG, triglyceride; TC, total cholesterol; HDL-C, high density lipoprotein cholesterol; LDL-C, low density lipoprotein cholesterol; Glu, blood glucose; BUN, blood urea nitrogen; Cr, creatinine; UA, uric acid, * P < 0.05, ** P < 0.01.

A power analysis revealed that, for α = 0.05, our study had a power to detect differences across cases and controls of 0.85 for the C-carrying genotype of T1880C, 0.93 for the A-carrying genotype of C602A, and 0.89 for the C-carrying genotype of T1559C. The genotype frequencies of C602A were significantly different (P = 0.005) between EH and NT groups, and the C-allelic frequency was also significantly different (P = 0.001; Table 3). A comparison of dominant versus recessive models (CC + CA vs. AA, or AA + CA vs. CC) revealed that significant differences persisted (P = 0.004 and P = 0.02 for dominant and recessive models, respectively). Interestingly, when subgrouped by sex, these indexes were significantly different in male NT and EH groups, but not in female NT and EH groups. The additive model of the T1559C genotype was not different between EH and NT groups (P = 0.39), but it was different between male EH and NT groups (P = 0.046) and female EH and NT groups (P = 0.045). The CC + TC versus TT frequency of T1559C was also different in a recessive model of male EH and NT groups (P = 0.02). Furthermore, there was a significant interaction between E-selectin and gender (Pinteraction = 0.02 for C602A and 0.04 for T1559C), but there was no significant interaction between the E-selectin genotypes and other indexes. As for the SNP T1880C, neither genotype distribution nor allelic frequency was obviously different between hypertensive patients and healthy normotensive subjects.

**Table 3:** Genotype and allele distribution in normotensive and essential hypertensive participants

		Total			Male			Female		
				
SNPs	Genotype	NT(n = 495)	EH(n = 490)	P	NT(n = 293)	EH(n = 316)	P	NT(n = 202)	EH(n = 174)	P
**T1880C**										
**Additive**	TT	2(0.4%)	3(0.6%)	0.44	2(0.7%)	3(0.9%)	0.65	0(0.0%)	0(0.0%)	0.38
	TC	48(9.7%)	37(7.6%)		29(9.9%)	25(7.9%)		19(9.4%)	12(6.9%)	
	CC	445(89.9%)	450(91.8%)		262(89.4%)	288(91.1%)		183(90.6%)	162(93.1%)	
**Dominant**	TT + TC	50(10.1%)	40(8.9%)	0.29	31(10.6%)	28(8.9%)	0.47	19(9.4%)	12(6.9%)	0.39
	CC	445(89.9%)	450(91.1%)		262(89.4%)	288(91.1%)		183(90.6%)	162(93.1%)	
	OR		1.26			1.22			1.40	
	95% CI		0.82-1.96			0.71-2.08			0.66-2.98	
**Recessive**	CC + TC	493(99.6%)	487(99.4%)	0.65	291(99.3%)	313(99.1%)	0.72	202(100%)	174(100%)	-
	TT	2(0.4%)	3(0.6%)		2(0.7%)	3(0.9%)		0	0	
	OR		1.52			1.40				-
	95% CI		0.25-9.13			0.23-8.41			-	
**Allele**	T	52(5.3%)	43(4.4%)	0.37	33(5.6%)	31(4.9%)	0.57	19(4.7%)	12(3.4%)	0.39
	C	938(94.7%)	937(95.6%)		553(94.4%)	601(95.1%)		385(95.3%)	336(96.6%)	
	OR		1.21			1.16			0.38	
	95% CI		0.80-1.83			0.70-1.91			0.66-2.89	
**C602A**										
**Additive**	CC	0(0.0%)	5(1.0%)	0.005	0(0.0%)	4(1.3%)	0.01	0(0.0%)	1(0.6%)	0.33
	CA	21(4.2%)	38(7.8%)		11(3.8%)	25(7.9%)		10(5.0%)	13(10.6%)	
	AA	474(95.8%)	447(91.2%)		282(96.2%)	287(90.8%)		192(95.0%)	160(92.0%)	
**Dominant**										
	CC + CA	21(4.2%)	43(8.8%)	0.004	11(3.8%)	29(9.2%)	0.007	10(5.0%)	14(8.0%)	0.22
	AA	474(95.8%)	447(91.2%)		282(96.2%)	287(90.8%)		192(95.0%)	160(92.0%)	
	OR		0.461			0.386			0.595	
	95% CI		0.269-0.788			0.189-0.788			0.257-1.376	
**Recessive**										
	AA + CA	495(100%)	485(99.0%)	0.02	293(100%)	312(98.7%)	0.05	202(100%)	173(99.4%)	0.28
	CC	0(0%)	5(1.0%)		0(0%)	4(1.3%)		0(0%)	1(0.6%)	
	OR		1.010			1.013			1.006	
	95% CI		1.001-1.019			1.000-1.026			0.994-1.017	
**Allele**										
	C	21(2.1%)	48(4.9%)	0.001	11(1.9%)	33(5.2%)	0.002	10(2.5%)	15(4.3%)	0.16
	A	969(97.9%)	932(95.1%)		575(98.1%)	599(94.8%)		394(97.5%)	333(95.7%)	
	OR		0.421			0.347			0.563	
	95% CI		0.250-708			0.174-0.694			0.250-1.271	
**T1559C**										
**Additive**	TT	43(8.7%)	33(6.7%)	0.39	30(10.2%)	16(5.1%)	0.046	13(6.4%)	17(9.8%)	0.045
	TC	200(40.4%)	191(39.0%)		115(39.2%)	124(39.2%)		85(42.1%)	67(38.5%)	
	CC	252(50.9%)	266(54.3%)		148(50.5%)	176(55.7%)		104(51.5%)	90(51.7%)	
**Dominant**	TT + TC	243(49.1%)	224(45.7%)	0.29	145(49.5%)	140(44.3%)	0.20	98(48.5%)	84(48.3%)	0.96
	CC	252(50.9%)	266(54.3%)		148(50.5%)	176(55.7%)		104(51.5%)	90(51.7%)	
	OR		1.145			1.232			1.010	
	95% CI		0.892-1.471			0.895-1.694			0.673-1.515	
**Recessive**	CC + TC	452(91.3%)	457(93.3%)	0.25	263(89.8%)	300(95.0%)	0.02	189(93.6%)	157(90.2%)	0.23
	TT	43(8.7%)	33(6.7%)		30(10.2%)	16(5.0%)		13(6.4%)	17(9.8%)	
	OR		0.759			0.468			1.574	
	95% CI		0.474-1.217			0.249-0.877			0.742-3.341	
**Allele**	T	286(28.9%)	257(26.2%)	0.19	175(30.0%)	156(24.7%)	0.05	111(27.5%)	101(29.0%)	0.63
	C	704(71.1%)	723(73.8%)		415(70.0%)	476(75.3%)		293(72.5%)	247(71.0%)	
	OR		1.143			1.287			0.926	
	95% CI		0.938-1.393			0.999-1.657			0.674-1.274	

EH, essential hypertensive group; NT, normotensive group; OR, odds ratio; 95%CI, 95% confidence interval.

**Figure 1:**
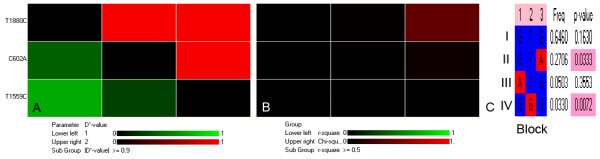
**Linkage disequilibrium analysis and haplotype block analysis of SNP T1880C, C602A and T1559C. **A. Linkage disequilibrium structure and the degree of linkage disequilibrium (D') for each SNP (T1880C, C602A and T1559C) pair. B. LD structure and the degree of linkage disequilibrium (r') for each SNP (T1880C, C602A and T1559C) pair. C. SNPs associating to hypertension and D' haplotype block. 1-T1880C; 2-C602A; 3-T1559C.

The variables height, weight, BMI, age, and genotype showed an association with blood pressure (P < 0.05); therefore, they were included in a stepwise logistic regression to study the possible combined effect of mutant C602A and T1559C alleles and other risk factors on hypertension. The remaining variables that were closely associated with BP were BMI, C602A, T1559C, height and weight after adjusting for age and sex (Table 4). In the haplotype-based case-control analysis, haplotypes were established in three representative common SNPs (Table 5). An association with hypertension was found in the linkage disequilibrium (LD) block (r2 > 0.5, D' > 0.9). C602A and T1559C in the block were significantly associated with hypertension (P = 0.033 and P = 0.007, respectively; Figure 1).

**Table 4:** Results of multiple logistic regression analysis: final significant variables in equation

Risk Factor	**Wald χ**^**2**^	P	OR	95% C.I
**BMI**	30.73	<0.01	0.80	0.74-0.87
**C602A**	5.69	<0.01	7.58	1.53-11.97
**T1559C**	6.12	<0.05	6.77	1.07-1.83
**Height**	9.51	<0.01	1.07	1.02-1.11
**Weight**	27.93	<0.01	0.92	0.90-0.95

BMI, body mass index; OR, odds ratio; 95%CI, 95% confidence interval.

**Table 5:** Haplotype analysis in normotensive and essential hypertensive participants

	Haplotype			Total			Male			Female		
				
	T1880C	C602A	T1559C	EH (n = 490)	NT (n = 495)	P	EH (n = 316)	NT (n = 293)	P	EH (n = 174)	NT (n = 202)	P
H1	G	T	G	0.66	0.64	0.43	0.66	0.63	0.17	0.64	0.66	0.60
H2	G	T	A	0.25	0.29	0.10	0.24	0.30	0.02	0.28	0.27	0.68
H3	A	T	G	0.04	0.05	0.28	0.04	0.05	0.34	0.03	0.04	0.40
H4	G	G	G	0.04	0.02	0.008	0.05	0.02	0.01	0.03	0.02	0.36
H5	G	G	A	0.008	0.002	0.16	0.006	0.003	0.09	0.009	0.004	0.46
H6	A	T	A	0.002	0.002	0.92	0.003	0.005	0.82	0.004	0.004	0.80

EH, essential hypertensive patients; NT, normotensive subjects.

For the total population and for males, the C-C-C haplotype (established from T1880C, C602A and T1559C) was significantly higher among essential hypertension patients than among control individuals as well as in hypertensive males and normotensive males (P = 0.008 and 0.012). The C-A-T haplotype was higher in the EH group than in the NT group (P = 0.015) among males. There were no significant differences in other haplotypes between the EH and NT groups in males, females, or over the entire population.

## Discussion

Genetics and environmental factors both play very important roles in the establishment and development of essential hypertension. Genetics is considered to be of clinical importance by physicians and researchers in the pathogenesis, diagnosis, treatment and prevention of hypertension. With the advent of the human genome project and the international HapMap project, SNPs have become increasingly prominent in the study of both multifactorial and multi-genomic diseases. E-selectin, the product of the SELE gene, is closely associated with hypertension, coronary heart diseases and atherosclerosis, and thus appears to be a risk factor for these conditions. The gene encoding E-selectin is more strongly expressed in the plasma of hypertensive patients than in NT subjects [13,14], and the level of E-selectin in sedentary, drug-treated hypertensives is significantly reduced after lifestyle intervention [15], confirming the association of E-selectin with hypertension. Moreover, Derzbach and colleagues found that the S128R variant of E-selectin is closely related to severe preeclampsia [16], and another study reported that the L554P variant increased the risk of BP in overweight people [7]. However, to our knowledge, the association of T1880C, C602A, T1559C E-selectin variants with essential hypertension has not been previously investigated.

In this study, we found that the frequency of the genotype C602A was significantly different between hypertensive patients and healthy normotensive subjects (P = 0.005). Moreover, the prevalence of the A allele was markedly higher in hypertensive patients than in healthy subjects (OR = 0.42, P = 0.001). Because a significant association of the SNP C602A with essential hypertension was obtained in a multivariate analysis after adjusting for confounding risk factors, including age, body weight and height, the A allele of C602A may be an independent risk factor for hypertension. Grouping the subjects in this study by sex revealed that males exhibited a similar association with essential hypertension, whereas females did not, possibly indicating that C602A is more significantly associated with hypertension in males. On the basis of these results, we conclude that C602A is associated with hypertension. The frequency of the T1559C SNP was also significantly different between normotensive subjects and hypertensive patients when grouped by sex. A multivariate analysis, applied as described above, also indicated an association between T1559C and hypertension.

Haplotype analysis is considered a good method for studying the genetics of complex diseases while avoiding problems incurred by multiple testing. In this study, two haplotypes of E-selectin were significantly associated with essential hypertension. Moreover, a power analysis revealed that, for α = 0.05, our study had the power to detect differences across cases and controls of 0.93 for the A-carrying genotype of C602A, and 0.89 for the C-carrying genotype of T1559C. Furthermore, the distribution of all polymorphisms was in Hardy-Weinberg equilibrium (data not shown), suggesting that the results of this study are unlikely to be biased by population stratification or admixture for essential hypertension. Taken together, our data strongly suggest that C602A and T1559C are associated with essential hypertension. The potential mechanism by which the common SNPs (C602A and T1559C) might contribute to essential hypertension remains unknown. Because these two SNPs are located in exons and the variants exhibit changes in amino acidic residues, it is possible that the resulting proteins promote enhanced phagocyte and/or adhesive activity of the endothelium and provoke inflammation in the vascular wall--a characteristic of hypertension [17]. Endothelium damage and dysfunction and disturbance of endothelially derived vasomodulatory factors may be another explanation [18]. However, additional studies will be required.

The minor allele frequencies (MAF) of the three SNPs in this study, T1880C(5.3%):C602A(2.1%):T1559C(28.9%), differed from the values reported in the NCBI SNP database T1880C(1.1%):C602A(1.1%):T1559C(32.6%) submitted by HapMap project (http://www.ncbi.nlm.nih.gov/projects/SNP/snp_viewTable.cgi?pop=1410). Because population distribution and sample size are very important determinants of MAF, it is likely that the small sample size in the HapMap project (n = 45) compared to the larger sample size (n = 495) in our study, and differences in the population distribution (individuals living in the residential community at Beijing Normal University in the HapMap project vs. Beijingers in our study) are responsible for the MAF difference between the HapMap project and our study.

The present population-based, case-control study also showed significant gender-specific (male only) differences in genetic markers between essential hypertension patients and control individuals. Moreover, we noted a significant gene-by-gender interaction with BP traits in this study. Several studies have reported gender-specific effects of gene variants and gene-by-gender interactions in human hypertension [19-22]. These data support our findings. One potential mechanism by which two common SNPs (C602A and T1559C) might contribute to essential hypertension in males is that these two variants increase susceptibility to endothelial dysfunction, inflammation and atherosclerosis. Although this is consistent with the fact that males have higher incidences of essential hypertension than females [23], more studies were needed. As for SNP T1880C, both genotypes and alleles exhibited similar frequencies in hypertensive patients and healthy normotensive subjects, even after adjusting for age and sex.

Certain limitations in the population under study lead to an increased risk of overestimating the statistical significance of MAFs. Accordingly, to enhance the reliability of conclusions, future studies should strive to achieve a more representative population distribution, larger sample sizes, a wider age distribution, and different blood pressure range subgroups.

## Conclusion

This study provides new evidence in support of an association between E-selectin and hypertension, showing that C602A and T1559C polymorphisms of E-selectin are novel candidate essential hypertension-associated SNPs, whereas T1880C is not. Future studies might explore these associations in different ethnic groups and investigate the mechanisms by which the C602A and T1559C polymorphisms of E-selectin might lead to hypertension.

## Abbreviations

SNP: single nucleotide polymorphism; BMI: body mass index; TC: total cholesterol; LDL-C: low density lipoprotein cholesterol; SBP: systolic blood pressure; DBP: diastolic blood pressure; EH: essential hypertensive; NT: normotensive; JNC: the seventh report of the joint national committee on prevention, detection, evaluation, and treatment of high blood pressure linkage disequilibrium: LD; MAF: minor allele frequency.

## Competing interests

The authors declare that they have no competing interests.

## Authors' contributions

ZGW carried out the molecular genetic studies and drafted the manuscript. YL, JLL, KL, YQL and QLN carried out the subjects screening and sampling. JW, SJW and ZSW participated in the design of the study and performed the statistical analysis. All authors read and approved the final manuscript.

## Pre-publication history

The pre-publication history for this paper can be accessed here:

http://www.biomedcentral.com/1471-2350/11/127/prepub
